# Testing for Coccidioidomycosis among Community-Acquired Pneumonia Patients, Southern California, USA^1^

**DOI:** 10.3201/eid2404.161568

**Published:** 2018-04

**Authors:** Sara Y. Tartof, Kaitlin Benedict, Fagen Xie, Gunter K. Rieg, Kalvin C. Yu, Richard Contreras, Jonathan Truong, Kimberlee Fong, Hung Fu Tseng, Steven J. Jacobsen, Rajal K. Mody

**Affiliations:** Kaiser Permanente Southern California, Pasadena, California, USA (S.Y. Tartof, F. Xie, R. Contreras, H.F. Tseng, S.J. Jacobsen);; Centers for Disease Control and Prevention, Atlanta, Georgia, USA (K. Benedict, R.K. Mody);; Southern California Permanente Medical Group, Pasadena (G.K. Rieg, K.C. Yu, J. Truong, K. Fong)

**Keywords:** coccidioidomycosis, community-acquired pneumonia, pneumonia, California, fungi, bacteria, respiratory infections, United States

## Abstract

We conducted a cohort study to identify characteristics associated with testing for, and testing positive for, coccidioidomycosis among patients with community-acquired pneumonia in southern California, USA. Limited and delayed testing probably leads to underdiagnosis among non-Hispanic black, Filipino, or Hispanic patients and among high-risk groups, including persons in whom antimicrobial drug therapy has failed.

The public health impact of coccidioidomycosis (Valley fever) in the United States is increasing. The causative fungus, *Coccidioides*, is endemic to the southwestern and western United States. In 2015, California reported 3,015 cases, ≈25% of all US cases (*1*,*2*). In areas of Arizona where coccidioidomycosis is highly endemic, the disease might be responsible for 15%–29% of community-acquired pneumonia (CAP) cases; however, in some studies, <15% of CAP patients are tested, suggesting that the disease is underrecognized, even in endemic areas (*3*–*5*). Testing practices for CAP patients in southern California have not been well documented. Therefore, we determined the proportion of CAP patients who were tested, the proportion who tested positive, and clinical factors associated with being tested and having confirmed coccidioidomycosis among patients enrolled in the Kaiser Permanente Southern California (KPSC) healthcare system in 2011.

## The Study

KPSC is an integrated healthcare organization with ≈4.4 million members who are representative of the socioeconomic and racial/ethnic diversity of the area’s population (*6*). KPSC uses electronic health records (EHRs) to integrate medical information from all care and laboratory settings. We included all KPSC patients meeting membership criteria who had CAP diagnosed and received treatment for CAP as outpatients (Technical Appendix Table 1). Information about each patient’s medical history during 2001–2011 was based on International Classification of Disease, Ninth Revision (ICD-9), codes retrieved from EHRs.

We searched CAP patient EHRs to identify coccidioidomycosis laboratory testing from all care settings (Technical Appendix Table 1). We sought documentation of a coccidioidomycosis-related ICD-9 code (114.X) in any encounter type from 1 week before to 1 year after the sample collection date for patients with confirmed coccidioidomycosis cases. We excluded patients having received an ICD-9 coccidioidomycosis diagnosis before 2011 and those who had a hospitalization during the 2 weeks before CAP diagnosis.

To identify factors for multivariable modeling, we used χ^2^ and *t*-tests (statistical significance defined as p<0.2), clinical knowledge, and a backward selection algorithm testing for interaction terms. We conducted analyses in SAS version 9.3 (SAS Institute, Cary, NC, USA).

After exclusions, the final cohort consisted of 33,756 patients (Technical Appendix Table 2). Among patients with CAP, 2,061 (6%) were tested for coccidioidomycosis within 1 year of CAP diagnosis. A median of 6 (mean 46) days and a median of 2 (mean 5) clinic encounters elapsed between the index CAP date (i.e., the date the CAP ICD-9 code was first used) and the first order for a coccidioidomycosis test. Among patients who initially tested negative, 5% had a repeat test within 30 days and 8% within 90 days.

In adjusted analyses, testing for coccidioidomycosis was less likely among female patients and among Hispanic patients who survived 1 year after the index CAP date (compared with surviving non-Hispanic whites) (Technical Appendix Table 2). Pulmonary clinics were most likely to test for coccidioidomycosis. Increasing numbers of healthcare encounters involving a CAP ICD-9 code, inpatient visits, chest radiograph orders, and antimicrobial drug prescriptions in the year after CAP diagnosis increased the odds of being tested for coccidioidomycosis. Patients whose race/ethnicity was Filipino, Hispanic, American Indian/Alaska Native multiple, other, or unknown who died (from any cause) had increased odds of being tested for coccidioidomycosis compared with surviving non-Hispanic whites.

Of the 2,061 CAP patients tested for coccidioidomycosis, 377 (18%) were positive by any test; of these, 45 (12%) had >1 previous negative test before testing positive, and 172 (46%) were confirmed by complement fixation or immunodiffusion. Among those who tested positive by both IgG and IgM enzyme immunoassay (EIA), 88% were confirmed by complement fixation or immunodiffusion; only 10% of IgG-positive results and 7% of IgM-positive results were confirmed (Technical Appendix Table 3).

In adjusted analyses, female sex was associated with reduced odds of testing positive (adjusted odds ratio [aOR] 0.60 [95% CI 0.42–0.86]). Persons of Filipino ethnicity (aOR 3.56 [95% CI 1.57–8.08]), non-Hispanic black race (aOR 2.78 [95% CI 1.50–5.12]), and Hispanic ethnicity (aOR 1.83 [95% CI 1.23–2.73) were more likely to test positive than were non-Hispanic whites. Kern County residents were more likely to test positive than Los Angeles County residents (aOR 2.48 [95% CI 1.56–3.95]) (Technical Appendix Table 4). Having antimicrobial drugs prescribed >2 times (in addition to the treatment-defining CAP diagnosis) from 1 week before the first CAP visit to the first coccidioidomycosis test (aOR 4.57 [95% CI 1.29–16.12]) and having chest radiographs within 1 year after CAP diagnosis (aOR 2.30 [95% CI 1.54–3.45]) were associated with increased odds of testing positive.

## Conclusions

We assessed testing practices for coccidioidomycosis among patients with CAP in southern California and found that only 6% of CAP patients were tested, of whom 18% were coccidioidomycosis-positive by any test and 8% by confirmatory testing. Further, our data highlight delayed testing for some patients, low rates of retesting, and opportunities to reduce unnecessary antibiotic use.

In addition to low overall testing rates, we detected substantial testing delays, suggested by much higher estimated mean (compared with median) time to testing. We might underestimate delays because patients might have had CAP-related visits before the study period began. Delays in testing have been noted previously but were shorter among persons who knew about the disease before seeking healthcare, suggesting a benefit of community awareness (*7*).

Delays in testing affect healthcare use. CAP patients tested for coccidioidomycosis were more likely to have received multiple courses of antimicrobial drugs, experienced more inpatient admissions for CAP, and received more chest radiographs than CAP patients who were not tested, suggesting substantial resource utilization and possible worsening of symptoms before coccidioidomycosis was considered. Further, patients with confirmed coccidioidomycosis were more likely to have received >2 additional antimicrobial drug prescriptions between CAP diagnosis and their first coccidioidomycosis test. Other studies have described high rates of initial and subsequent antimicrobial treatment among coccidioidomycosis patients in Arizona (*5*,*7*).

Patients of Filipino, Hispanic, non-Hispanic black, and American Indian/Alaska Native or multiple, other, or unknown race/ethnicity who died had ≈8, 2, 2, and 3 times the odds of being tested for coccidioidomycosis, respectively, compared with surviving non-Hispanic whites. Although we could only capture all-cause mortality, the high probability for testing among patients who do not survive suggests possible progression of severe disease before consideration of coccidioidomycosis. Additionally, non-Hispanic black and Filipino patients with CAP had greater odds than non-Hispanic whites for having coccidioidomycosis. Historically, non-Hispanic black and Filipino patients have been identified as having increased risk for severe or disseminated coccidioidomycosis compared with other racial/ethnic groups (*8*–*11*). Unfortunately, we were unable to control for exposure-related factors, such as occupation, which might correlate with race/ethnicity.

Experts at the University of Arizona suggest that patients who initially have a negative serologic test should be retested within 2 months because serologic tests can be negative early in the course of infection (*12*). In our cohort, 12% of patients with any positive coccidioidomycosis test had previous negative tests. However, few CAP patients (8%) who tested negative were retested. Thus, increased awareness of repeat testing for those with persistent symptoms might be warranted. However, EIA testing has limitations; although it is widely used because it is faster and requires less technical expertise than complement fixation or immunodiffusion, the specificity is low. Having a positive EIA test result for IgG or IgM alone in our study correlated very poorly with positive confirmatory testing.

In conclusion, limited testing for coccidioidomycosis likely precludes accurate assessment of the overall frequency of the disease among CAP patients. Physician and community education might improve overall detection and result in earlier detection, which could be beneficial in decreasing overuse of antimicrobial drugs, reducing time and resources spent seeking other diagnoses, and improving monitoring for coccidioidomycosis complications.

Technical Appendix Case definitions, inclusion and exclusion criteria, patient characteristics, and test results for study of coccidioidomycosis testing among patients with community-acquired pneumonia, southern California, USA.

## References

[1] Marsden-Haug N, Goldoft M, Ralston C, Limaye AP, Chua J, Hill H, et al. Coccidioidomycosis acquired in Washington State. Clin Infect Dis. 2013;56:847–50. 10.1093/cid/cis1028 23223598

[2] CDC. Notice to readers: final 2015 reports of nationally notifiable infectious diseases and conditions. MMWR Morb Mortal Wkly Rep. 2016;65:1306–21. 10.15585/mmwr.mm6546a9 27880744

[3] Chang DC, Anderson S, Wannemuehler K, Engelthaler DM, Erhart L, Sunenshine RH, et al. Testing for coccidioidomycosis among patients with community-acquired pneumonia. Emerg Infect Dis. 2008;14:1053–9. 10.3201/eid1407.070832 18598625 PMC2600364

[4] Kim MM, Blair JE, Carey EJ, Wu Q, Smilack JD. Coccidioidal pneumonia, Phoenix, Arizona, USA, 2000-2004. Emerg Infect Dis. 2009;15:397–401. 10.3201/eid1563.081007 19239751 PMC2681119

[5] Valdivia L, Nix D, Wright M, Lindberg E, Fagan T, Lieberman D, et al. Coccidioidomycosis as a common cause of community-acquired pneumonia. Emerg Infect Dis. 2006;12:958–62. 10.3201/eid1206.060028 16707052 PMC3373055

[6] Koebnick C, Langer-Gould AM, Gould MK, Chao CR, Iyer RL, Smith N, et al. Sociodemographic characteristics of members of a large, integrated health care system: comparison with US Census Bureau data. Perm J. 2012;16:37–41. 10.7812/TPP/12-031 23012597 PMC3442759

[7] Tsang CA, Anderson SM, Imholte SB, Erhart LM, Chen S, Park BJ, et al. Enhanced surveillance of coccidioidomycosis, Arizona, USA, 2007-2008. Emerg Infect Dis. 2010;16:1738–44. 10.3201/eid1611.100475 21029532 PMC3294516

[8] Crum NF, Lederman ER, Stafford CM, Parrish JS, Wallace MR. Coccidioidomycosis: a descriptive survey of a reemerging disease. Clinical characteristics and current controversies. Medicine (Baltimore). 2004;83:149–75. 10.1097/01.md.0000126762.91040.fd 15118543

[9] Rosenstein NE, Emery KW, Werner SB, Kao A, Johnson R, Rogers D, et al. Risk factors for severe pulmonary and disseminated coccidioidomycosis: Kern County, California, 1995-1996. Clin Infect Dis. 2001;32:708–15. 10.1086/319203 11229838

[10] Smith CE, Beard RR, Whiting EG, Rosenberger HG. Varieties of coccidioidal infection in relation to the epidemiology and control of the diseases. Am J Public Health Nations Health. 1946;36:1394–402. 10.2105/AJPH.36.12.1394 20278046 PMC1624510

[11] Gifford M. Coccidioidomycosis, Kern County [cited 2016 Jul 12]. http://kerncountyvalleyfever.com/wp-content/uploads/2013/04/LibraryCocci-Article-1939.pdf

[12] Valley Fever Center for Excellence. Valley fever (coccidioidomycosis) tutorial for primary care professionals [cited 2016 Jul 12]. http://vfce.arizona.edu/sites/vfce/files/tutorial_for_primary_care_professionals.pdf

