# Supplementary material for: Testing for Coccidioidomycosis among Community-Acquired Pneumonia Patients, Southern California, USA
Source: Emerg Infect Dis. 2018 Apr;24(4):779–81. doi: 10.3201/eid2404.161568 (PMC5875278; doi:10.3201/eid2404.161568)
Supplement: Technical Appendix — Case definitions, inclusion and exclusion criteria, patient characteristics, and test results for study of coccidioidomycosis testing among patients with community-acquired pneumonia, southern California, USA. [file 16-1568-Techapp-s1.pdf]

Article DOI: https://doi.org/10.3201/eid2404.161568

Testing for Coccidioidomycosis among Community-Acquired Pneumonia Patients, Southern California, USA

Technical Appendix

Technical Appendix Table 1. Case Definitions and Criteria for Inclusion and Exclusion for Conditions of Interest

Condition	Case Definition	Details
Community-Acquired Pneumonia	1) Select International Classification of Diseases, Ninth Revision, Clinical Modification (ICD-9) diagnosis codes	• 480.9 (viral pneumonia, unspecified)<br>• 482.9 (bacterial pneumonia, unspecified)<br>• 485 (bronchopneumonia, organism unspecified)<br>• 486 (pneumonia, organism unspecified)
	2) Chest radiograph (identified by Current Procedural Terminology [CPT] code) within 2 weeks before to 4 weeks after the date the CAP ICD-9 code was first used	CPT code: 71010, 71020, 71021, 71022, 71035, 71101, or 71111
	3) Prescription for a systemic antibacterial antibiotic within 2 weeks before to 2 weeks after the date the CAP ICD-9 code was first used	
Community-Acquired Pneumonia Exclusion Criteria	Those with a hospitalization in the 2 weeks before CAP diagnosis (to minimize hospital-acquired infections) were excluded	
Community-Acquired Pneumonia Membership Criteria	Those KPSC patients with continuous enrollment for one year before their CAP diagnosis date (allowing a 31-d gap for administrative delays in membership renewal) were included	
Coccidioidomycosis	Positive test by enzyme immunoassay (EIA) for IgM or IgG, complement fixation (CF), or immunodiffusion (ID) from any source in the 1 y following the date the CAP ICD-9 code was first used	
Confirmed Coccidioidomycosis	Positive CF or ID test, or culture of *Coccidioides* or histological report identifying *Coccidioides* among patients with antibody testing.	
Coccidioidomycosis Exclusion Criteria	Patients with a coccidioidomycosis diagnosis (based on ICD-9 code 114.X) before 2011 were excluded	

Technical Appendix Table 2. Comparison of Characteristics of Patients Tested for Coccidioidomycosis and those not Tested among Patients Presenting with Community-Acquired Pneumonia (CAP) in 2011

Characteristic	Total CAP Patients	Patients Not-tested for Coccidioidomycosis* Total n = 31,695 (100%)	Patients Tested for Coccidioidomycosis* Total n = 2,061 (100%)	Odds Ratio	95% CI	Adjusted Odds Ratio[¥]	95% CI
Sex							
Female	17,086	16,094 (50.8%)	992 (48.1%)	0.90	0.82–0.98	**0.85**	**0.77–0.95**
Male	16,670	15,601 (49.2%)	1,069 (51.9%)	1.00	Ref.	1.000	Ref.
Age Group							
≤9 y	9,643	9,513 (30.0%)	130 (6.3%)	1.00	Ref.	1.00	Ref.
10–18 y	3,357	3,254 (10.3%)	103 (5.0%)	2.32	1.78–3.01	**3.08**	**2.32–4.09**
19–40 y	3,533	3,254 (10.3%)	279 (13.5%)	6.27	5.09–7.78	**7.81**	**5.56–10.96**
41–60 y	7,194	6,494 (20.5%)	700 (34.0%)	7.89	6.55–9.58	**9.85**	**7.12–13.63**
61–80 y	7,625	6,944 (21.9%)	681 (33.0%)	7.18	5.96–8.71	**7.51**	**5.38–10.49**
81+ years	2,404	2,236 (7.1%)	168 (8.2%)	5.50	4.36–6.95	**3.99**	**2.74–5.81**
Race/Ethnicity[£]							
Non-Hispanic White	14,135	13,038 (41.1%)	1,097 (53.2%)	1.00	Ref.		
Non-Hispanic Black	2,663	2,520 (8.0%)	143 (6.9%)	0.67	0.56–0.80		
Hispanic	11,931	11,351 (35.8%)	580 (28.1%)	0.61	0.55–0.67		
Asian/ Hawaiian/Pacific Islander	1,998	1,920 (6.10%)	78 (3.8%)	0.48	0.38–0.62		
Filipino	1,267	1,192 (3.8%)	75 (3.6%)	0.75	0.58–0.95		
American Indian or Alaska Native/Multiple/Other /Unknown (AI/AN/Other)	1,762	1,674 (5.3%)	88 (4.3%)	0.63	0.50–0.78		
Clinic Setting							
Emergency	5,029	4,743 (15.0%)	286 (13.9%)	1.00	Ref.	1.00	Ref.
Urgent care	4,918	4,349 (13.7%)	569 (27.6%)	2.17	1.87–2.52	**1.27**	**1.06–1.54**
Family or Internal medicine	12,619	11,717 (37.0%)	902 (43.8%)	1.28	1.12–1.47	**1.29**	**1.09–1.52**
Pediatrics	10,302	10,132 (32.0%)	170 (8.3%)	0.28	0.23–0.34	1.08	0.77–1.51
Pulmonary	165	116 (0.4%)	49 (2.4%)	7.01	4.88–9.93	**6.02**	**3.95–9.17**
Unknown or other clinics	723	638 (2.0%)	85 (4.1%)	2.21	1.70–2.84	0.78	0.56–1.07
County of Residence							
Los Angeles County	13,709	12,983 (41.0%)	726 (35.0%)	1.00	Ref.	1.00	Ref.
Kern County	1,454	815 (2.6%)	639 (31.0%)	14.02	12.34–15.93	**18.43**	**15.64–21.72**
Orange County	4,084	3,980 (12.6%)	104 (5.1%)	0.47	0.38–0.57	**0.34**	**0.27–0.43**
Riverside County	3,163	3,042 (9.6%)	121 (5.9%)	0.71	0.58–0.86	**0.60**	**0.49–0.74**
San Bernardino County	3,717	3,601 (11.4%)	116 (5.6%)	0.58	0.47–0.70	**0.45**	**0.37–0.56**
San Diego County	6,696	6,411 (20.2%)	285 (13.8%)	0.80	0.69–0.91	**0.66**	**0.57–0.78**
Ventura County	884	819 (2.6%)	65 (3.2%)	1.42	1.08–1.83	0.98	0.73–1.31
Other County**	49	44 (0.1%)	5 (0.2%)	2.03	0.70–4.68	1.22	0.43–3.48
History of DM	5,255	4,811 (15.2%)	444 (21.5%)	1.53	1.37–1.71	**0.69**	**0.60–0.79**
History of COPD	3,541	3,187 (10.1%)	354 (17.2%)	1.86	1.64–2.09	**0.63**	**0.53–0.74**
History of Asthma	10196	9,640 (30.4%)	556 (27.0%)	0.85	0.76–0.93	**0.82**	**0.73–0.93**
History of Neoplasm or cancer	4,188	3,799 (12.0%)	389 (18.9%)	1.71	1.52 - 1.92	0.89	0.77–1.03
History of HIV/AIDS	89	71 (0.2%)	18 (0.9%)	3.92	2.27–6.44	**3.39**	**1.86–6.16**
History of Tuberculosis	150	136 (0.4%)	14 (0.7%)	1.59	0.87–2.66		
Prescribed chemotherapy agents within one week before/on CAP diagnosis	72	61 (0.2%)	11 (0.5%)	2.79	1.39–5.08	1.29	0.56–2.95
Prescribed corticosteroid agents within one week before/on CAP diagnosis	6,876	6,450 (20.4%)	426 (20.7%)	1.02	0.91–1.14		

Characteristic	Total CAP Patients	Patients Not-tested for Coccidioidomycosis* Total n = 31,695 (100%)	Patients Tested for Coccidioidomycosis* Total n = 2,061 (100%)	Odds Ratio	95% CI	Adjusted Odds Ratio[¥]	95% CI
Influenza-like illness within 4 weeks before/on CAP diagnosis	3,825	3,614 (11.4%)	211 (10.2%)	0.89	0.76–1.02		
Pregnant during any time in 2011(female CAP patient only)[‡]	103	97 (0.6%)	6 (0.6%)	1.00	0.39–2.11		
Died before January 1, 2013	1,374	1,140 (3.6%)	234 (11.4%)	3.43	2.95–3.98		
Total number of encounters (outpatient and emergency) within 4 weeks before CAP diagnosis							
0	15,898	15,113 (47.7%)	785 (38.1%)	1.00	Ref.	1.00	Ref.
1	9,705	9,123 (28.8%)	582 (28.2%)	1.23	1.10–1.37	**1.16**	**1.02–1.32**
2	4,413	4,095 (12.9%)	318 (15.4%)	1.50	1.31–1.71	**1.22**	**1.04–1.43**
3	1,865	1,706 (5.4%)	159 (7.7%)	1.79	1.50–2.14	1.21	0.98–1.50
4+	1,875	1,658 (5.2%)	217 (10.5%)	2.52	2.15–2.95	**1.25**	**1.02–1.52**
Total number of CAP follow up encounters within one year after CAP diagnosis							
0	21,544	20,946 (66.1%)	598 (29.0%)	1.00	Ref.	1.00	Ref.
1	7,212	6,706 (21.2%)	506 (24.6%)	2.64	2.34–2.98	**2.05**	**1.66–2.52**
2	2,635	2,313 (7.3%)	322 (15.6%)	4.88	4.23–5.62	**3.17**	**2.46–4.11**
3	1,142	912 (2.9%)	230 (11.2%)	8.83	7.47–10.41	**5.72**	**4.24–7.72**
4+	1,223	818 (2.6%)	405 (19.7%)	17.34	15.01 –20.03	**9.02**	**6.46–12.60**
Follow up emergency services within one year after CAP diagnosis	2,102	1,703 (5.4%)	399 (19.4%)	4.23	3.75–4.76	**0.75**	**0.61–0.92**
Admitted to hospital (inpatient) within one year after CAP diagnosis	2,492	1,974 (6.2%)	518 (25.1%)	5.06	4.53–5.64	**1.52**	**1.25–1.85**
x-ray ordered within one year follow up visits after CAP diagnosis	9,673	8,382 (26.5%)	1,291 (62.6%)	4.66	4.25–5.12	**1.34**	**1.10–1.62**
Total number of times prescribed corticosteroid agents within one week before/on follow-up visits within one year after CAP diagnosis							
0	31,283	29,631 (93.5%)	1,652 (80.2%)	1.00	Ref.	1.00	Ref.
1	1,974	1,706 (5.4%)	268 (13.0%)	2.82	2.45–3.23	1.04	0.87–1.26
2	355	253 (0.8%)	102 (5.0%)	7.23	5.69–9.12	1.38	1.00–1.91
3	98	75 (0.2%)	23 (1.1%)	5.50	3.37–8.65	0.63	0.34–1.17
4+	46	30 (0.1%)	16 (0.8%)	9.57	5.08–17.34	0.95	0.42–2.14
Total number of times prescribed additional antibiotic agents within one week before/on follow-up visits within one year after CAP diagnosis[¢]							
0	29,248	27,865 (87.9%)	1,383 (67.1%)	1.00	Ref.	1.00	Ref.
1	3,607	3,152 (9.9%)	455 (22.1%)	2.91	2.60–3.25	**1.36**	**1.15–1.60**
2	669	523 (1.7%)	146 (7.1%)	5.63	4.63–6.79	**1.62**	**1.23–2.14**
3	161	108 (0.3%)	53 (2.6%)	9.89	7.04–13.72	**2.10**	**1.31–3.35**
4+	71	47 (0.2%)	24 (1.2%)	10.29	6.18–16.70	**2.02**	**1.04–3.83**
Race/Ethnicity * Death							
Non-Hispanic White + Death (N)						1.00	Ref.
Non-Hispanic White + Death (Y)						**1.79**	**1.40–2.27**
Non-Hispanic Black + Death (Y)						0.89	0.41–1.94
Hispanic + Death (Y)						**1.91**	**1.30–2.81**
Asian/ Hawaiian/Pacific Islander + Death (Y)						0.89	0.31–2.60

Characteristic	Total CAP Patients	Patients Not-tested for Coccidioidomycosis* Total n = 31,695 (100%)	Patients Tested for Coccidioidomycosis* Total n = 2,061 (100%)	Odds Ratio	95% CI	Adjusted Odds Ratio¥	95% CI
Filipino + Death (Y)						**7.79**	**3.60–16.85**
AI/AN/Other + Death (Y)						**3.12**	**1.30–7.54**
Non-Hispanic Black + Death (N)						0.86	0.69–1.07
Hispanic + Death (N)						**0.81**	**0.71–0.93**
Asian/ Hawaiian/Pacific Islander + Death (N)						0.95	0.73–1.24
Filipino + Death (N)						0.89	0.66–1.20
AI/AN/Other + Death (N)						0.86	0.65–1.12

£Put back into the final model although it was eliminated by the model backward selection algorithm
‡Not included in the multivariable analysis because it only applied to females
*Tested within 1 y after CAP diagnosis
**Other County includes: Alameda County, Anderson County, Burlington County, Butte County, Carver County, Clark County, Clearwater County, Contra Costa County, Fresno County, Imperial County, Kauai County, Kootenai County, La Paz County, Merced County, Mohave County, Sacramento County, Santa Barbara County, Santa Clara County, Shasta County, Tulare County
¢All patients received at least one course of antibiotics; prescription of a systemic antibiotic from 2 weeks before to 4 weeks after the index date was one component of the CAP patient definition
¥Gray colored cells indicate variables that were not included in adjusted analyses due to P-value >0.2 in unadjusted analyses, or were not considered confounders a priori

Technical Appendix Table 3. Enzyme Immunoassay Testing for Coccidioidomycosis among Community-Acquired Pneumonia Patients Presenting in 2011, by Confirmatory Complement Fixation or Immunodiffusion Test

	Complement Fixation or Immunodiffusion*			
Enzyme Immunoassay (EIA)	Positive, no. (%)	Negative, no. (%)	Tested, unknown, no. (%)	Not tested, no. (%)
Positive by IgG + IgM (n = 170)	149 (87.65)	19 (11.18)	0 (0.00)	2 (1.18)
Positive by IgG Only (n = 71)	7 (9.86)	61 (85.92)	1 (1.41)	2 (2.82)
Positive by IgM Only (n = 129)	9 (6.98)	118 (91.47)	0 (0.00)	2 (1.55)
No EIA (n = 7)	7 (100.00)	0 (0.00)	0 (0.00)	0 (0.00)
Total (n = 377)	172 (45.62)**	198 (52.52)	1 (0.27)	6 (1.59)

*Tests performed within one year following a positive IgG and/or IgM test
**Of the 172 patients with confirmatory testing by complement fixation and/or immunodiffusion, 17 also had a positive culture or histopathology for Coccidioides.

Technical Appendix Table 4. Comparison of Characteristics of Patients with Confirmed Coccidioidomycosis versus those Testing Negative among Community-Acquired Pneumonia (CAP) Patients Presenting in 2011 who were Tested for Coccidioidomycosis

Characteristic	Total Patients Tested for Coccidioidomycosis	Patients Tested for Coccidioidomycosis with Negative Test Total n = 1889 (100%)	Patients with Confirmed Coccidioidomycosis[a] Total n = 172 (100%)	Odds Ratio	95% CI	Adjusted Odds Ratio[¥]	95% CI
Sex[£]							
Female	992	932 (49.3%)	60 (34.9%)	0.55	0.40–0.76	0.60	**0.42–0.86**
Male	1,069	957 (50.7%)	112 (65.1%)	1.00	Ref.	1.00	Ref.
Age Group							
≤9 y	130	125 (6.6%)	5 (2.9%)	1.00	Ref.	1.00	Ref.
10–18 y	103	87 (4.6%)	16 (9.3%)	4.60	1.62–13.0	6.71	**2.28–19.70**
19–40 y	279	231 (12.2%)	48 (27.9%)	5.20	2.02–13.38	9.96	**2.64–37.54**
41–60 y	700	629 (33.3%)	71 (41.3%)	2.82	1.12–7.13	8.38	**2.25–31.25**
61–80 y	681	651 (34.5%)	30 (17.4%)	1.15	0.49–3.03	5.95	**1.52–23.33**
81+ years	168	166 (8.8%)	2 (1.2%)	0.30	0.06–1.58	2.31	0.33–16.39
Race/Ethnicity							
Non-Hispanic White	1,097	1038 (55.0%)	59 (34.3%)	1.00	Ref.	1.00	Ref.
Non-Hispanic Black	143	124 (6.6%)	19 (11.1%)	2.70	1.56–4.67	2.78	**1.50–5.12**
Hispanic	580	509 (27.0%)	71 (41.3%)	2.45	1.71–3.52	1.83	**1.23–2.73**
Asian/ Hawaiian/Pacific Islander	78	70 (3.7%)	8 (4.7%)	2.01	0.92–4.37	1.78	0.77–4.12
Filipino	75	65 (3.4%)	10 (5.8%)	2.71	1.32–5.54	3.56	**1.57–8.08**
American Indian or Alaska Native/ Multiple /Other /Unknown	88	83 (4.4%)	5 (2.9%)	1.06	0.41–2.71	0.85	0.32 –2.29
Clinic Setting							
Emergency	286	278 (14.7%)	8 (4.7%)	1.00	Ref.	1.00	Ref.
Urgent care	569	502 (26.6%)	67 (39.0%)	4.64	2.20–9.80	1.87	0.81–4.32
Family and Internal medicine	902	837 (44.3%)	65 (37.8%)	2.70	1.28–5.70	1.50	0.67 - 3.40
Pediatrics	170	154 (8.2%)	16 (9.3%)	3.61	1.51–8.63	2.61	0.68–9.99
Pulmonary	49	48 (2.5%)	1 (0.6%)	0.72	0.09–5.92	0.79	0.09–6.92
Unknown or other clinics	85	70 (3.7%)	15 (8.7%)	7.45	3.04–18.26	4.04	**1.46–11.20**
County of Residence							
Los Angeles County	726	685 (36.3%)	41 (23.8%)	1.00	Ref.	1.00	Ref.
Kern County	639	541 (28.6%)	98 (57.0%)	3.03	2.07–4.43	2.48	**1.56–3.95**
Orange County	104	100 (5.3%)	4 (2.3%)	0.67	0.23–1.91	0.77	0.25–2.34
Riverside County	121	109 (5.8%)	12 (7.0%)	1.84	0.94–3.61	1.98	0.95–4.14
San Benardino County	116	112 (5.9%)	4 (2.3%)	0.60	0.21–1.70	0.62	0.21–1.84
San Diego County	285	275 (14.6%)	10 (5.8%)	0.61	0.30–1.23	0.78	0.36–1.69
Ventura County	65	62 (3.3%)	3 (1.7%)	0.81	0.24–2.69	0.83	0.22–3.08
Other County**	5	5 (0.3%)	0 (0.0%)				
History of DM	444	427 (22.6%)	17 (9.9%)	0.38	0.23–0.63	0.41	**0.23–0.73**
History of COPD	354	348 (18.4%)	6 (3.5%)	0.16	0.07–0.36	0.37	**0.15–0.91**
History of Asthma	556	529 (28.0%)	27 (15.7%)	0.48	0.31–0.73	0.68	0.42–1.09
History of Neoplasm or cancer	389	372 (19.7%)	17 (9.9%)	0.45	0.27–0.75	1.13	0.63–2.03
History of HIV/AIDS	18	17 (0.9%)	1 (0.6%)	0.65	0.09–4.87	0.72	0.08–6.28
History of Tuberculosis	14	13 (0.7%)	1 (0.6%)	0.84	0.11–6.49		
Prescribed chemotherapy agents within one week before/on CAP diagnosis	11	10 (0.5%)	1 (0.6%)	1.10	0.14–8.64		
Prescribed corticosteroid agents within one week before/on CAP diagnosis	426	393 (20.8%)	33 (19.2%)	0.90	0.61–1.34		

Characteristic	Total Patients Tested for Coccidioidomycosis	Patients Tested for Coccidioidomycosis with Negative Test Total n = 1889 (100%)	Patients with Confirmed Coccidioidomycosis[a] Total n = 172 (100%)	Odds Ratio	95% CI	Adjusted Odds Ratio[¥]	95% CI
Influenza-like illness within 4 weeks before/on CAP diagnosis	211	189 (10.0%)	22 (12.8%)	1.32	0.82–2.12	1.12	0.67–1.88
Pregnant during any time in 2011(female CAP patient only)[‡]	6	6 (0.6%)	0 (0.0%)				
Died before January 1, 2013	234	231 (12.2%)	3 (1.7%)	0.13	0.04–0.40	0.34	0.10–1.16
Total Number of encounters (outpatient and emergency) within 4 weeks before CAP diagnosis							
0	785	709 (37.5%)	76 (44.2%)	1.00	Ref.		
1	582	530 (28.1%)	52 (30.2%)	0.92	0.63–1.33		
2	318	291 (15.4%)	27 (15.7%)	0.87	0.55–1.37		
3	159	153 (8.1%)	6 (3.5%)	0.37	0.16–0.86		
4+	217	206 (10.0%)	11 (6.4%)	0.50	0.26–0.96		
Total Number of CAP follow-up encounters within one year after CAP diagnosis							
0	598	564 (29.9%)	34 (19.8%)	1.00	Ref.		
1	506	454 (24.0%)	52 (30.2%)	1.90	1.21–2.98		
2	322	290 (15.4%)	32 (18.6%)	1.83	1.11–3.03		
3	230	207 (11.0%)	23 (13.4%)	1.84	1.06–3.20		
4+	405	374 (19.8%)	31 (18.0%)	1.38	0.83–2.28		
Follow up emergency department visits within one year after CAP diagnosis	399	379 (20.1%)	20 (11.6%)	0.52	0.33–0.85	1.17	0.62–2.19
Inpatient stays within one year after CAP diagnosis	518	502 (26.6%)	16 (9.3%)	0.28	0.17–0.48	0.41	**0.21–0.80**
x-ray ordered within one year follow-up visits after CAP diagnosis	1,291	1,162 (61.5%)	129 (75.0%)	1.88	1.31–2.68	2.30	**1.54–3.45**
Total number of times prescribed corticosteroid agents during CAP visits from one week before/on initial CAP visit through first coccidioidomycosis test							
0	1,890	1,722 (91.2%)	168 (97.7%)	1.00	Ref.	1.00	Ref.
1+	171	167 (8.8%)	4 (2.3%)	0.25	0.09–0.67	0.29	**0.09–0.94**
Total number of times prescribed additional antibiotic agents during CAP visits from one week before/on initial CAP visit through first coccidioidomycosis test[¢]							
0	1,746	1,596 (84.5%)	150 (87.2%)	1.00	Ref.	1.00	Ref.
1	262	244 (12.9%)	18 (10.5%)	0.79	0.47–1.30	1.46	0.77–2.77
2+	53	49 (2.6%)	4 (2.3%)	0.87	0.46–3.68	4.57	**1.29–16.12**
Total number of encounters between initial CAP diagnosis and first coccidioidomycosis test							
0	659	588 (31.1%)	71 (41.3%)	1.00	Ref.	1.00	Ref.
1	324	293 (15.5%)	31 (18.0%)	0.88	0.56–1.37	1.39	0.82–2.33
2	243	219 (11.6%)	24 (14.0%)	0.91	0.56–1.48	1.49	0.84–2.64
3	172	160 (8.5%)	12 (7.0%)	0.62	0.33–1.17	1.09	0.53–2.27

Characteristic	Total Patients Tested for Coccidioidomycosis	Patients Tested for Coccidioidomycosis with Negative Test Total n = 1889 (100%)	Patients with Confirmed Coccidioidomycosis[a] Total n = 172 (100%)	Odds Ratio	95% CI	Adjusted Odds Ratio[¥]	95% CI
4+	663	629 (33.3%)	34 (19.8%)	0.45	0.29–0.68	0.95	0.54–1.65
Total days after initial CAP diagnosis that first coccidioidomycosis lab test was ordered[§]							
Mean (SD)	46.3 (84.4)	48.7 (86.5)	19.8 (48.7)				
Median (Range)	6 (0–365)	6.0 (0–365)	4 (0–287)				

[a]Confirmed by immunodiffusion and/or complement fixation test. Does not include the 3 patients identified by histopathology or culture
£Maintained in final model despite elimination by backward selection
‡Not included in the multivariate analysis; only applies to females
§ Tested by Wilcoxon rank sum test
*Tested within 1 y after CAP diagnosis
**Other County includes: Alameda County, Anderson County, Burlington County, Butte County, Carver County, Clark County, Clearwater County, Contra Costa County, Fresno County, Imperial County, Kauai County, Kootenai County, La Paz County, Merced County, Mohave County, Sacramento County, Santa Barbara County, Santa Clara County, Shasta County, Tulare County
¢All patients received at least one course of antibiotics; prescription of a systemic antibiotic from 2 weeks before to 4 weeks after the index date was one component of the CAP patient definition
¥Gray colored cells indicate variables that were not included in adjusted analyses due to P-value >0.2 in unadjusted analyses, or were not considered confounders a priori.
